# Skin microbiota of oxazolone-induced contact hypersensitivity mouse model

**DOI:** 10.1371/journal.pone.0276071

**Published:** 2022-10-20

**Authors:** Kuunsäde Mäenpää, Shuyuan Wang, Marit Ilves, Hani El-Nezami, Harri Alenius, Hanna Sinkko, Piia Karisola

**Affiliations:** 1 Human Microbiome Research Program, University of Helsinki, Helsinki, Finland; 2 School of Biological Sciences, University of Hong Kong, Pokfulam, Hong Kong; 3 Institute of Environmental Medicine (IMM), Karolinska Institutet, Stockholm, Sweden; Universidade Federal do Rio de Janeiro, BRAZIL

## Abstract

Contact allergy is a common skin allergy, which can be studied utilising contact hypersensitivity (CHS) animal model. However, it is not clear, whether CHS is a suitable model to investigate skin microbiota interactions. We characterised the effect of contact dermatitis on the skin microbiota and studied the biological effects of oxazolone (OXA) -induced inflammation on skin thickness, immune cell numbers and changes of the microbiota in CHS mouse model (n = 72) for 28 days. Through 16S rRNA gene sequencing we defined the composition of bacterial communities and associations of bacteria with inflammation. We observed that the vehicle solution of acetone and olive oil induced bacterial community changes on day 1, and OXA-induced changes were observed mainly on day 7. Many of the notably enriched bacteria present in the OXA-challenged positive group represented the genus *Faecalibaculum* which were most likely derived from the cage environment. Additionally, skin inflammation correlated negatively with *Streptococcus*, which is considered a native skin bacterium, and positively with *Muribacter muris*, which is typical in oral environment. Skin inflammation favoured colonisation of cage-derived faecal bacteria, and additionally mouse grooming transferred oral bacteria on the skin. Due to the observed changes, we conclude that CHS model could be used for certain skin microbiome-related research set-ups. However, since vehicle exposure can alter the skin microbiome as such, future studies should include considerations such as careful control sampling and statistical tests to account for potential confounding factors.

## Introduction

Contact allergy is a delayed hypersensitivity response affecting around 20% of the general population [1]. Skin or mucosal contact to the response-causing agent, such as nickel, causes rash, erythema, oedema and even blisters in the sensitised individuals [2]. The immunological mechanisms of contact allergy can be studied with the contact hypersensitivity (CHS) animal model [3]. It is a versatile tool that has been used to study specific questions on immune cell function, differentiation, and regulation [4–6]. However, it is not clear whether changes in the skin microbiome can be studied in CHS model.

Contact allergy begins with an exposure to a low-molecular weight chemical, a hapten, which becomes immunogenic after penetrating the skin and binding to cellular proteins [7] (Azeem et al 2020). In the CHS models, shaved mouse skin is exposed to the hapten, e.g. oxazolone (OXA), and hapten penetration is enhanced by mixing it with a vehicle solution, such as acetone and olive oil [8]. The immunogenic complexes are recognised by antigen-presenting cells, such as dendritic cells and Langerhans cells, which then uptake the complexes, migrate to skin-draining lymph nodes, and present the processed antigens to T cells [7]. The differentiated T cells reside in the skin-draining lymph-nodes as effector memory cells until next exposure to the same hapten. The re-exposure leads to proliferation and migration of effector memory cells to the site of exposure, and subsequently to inflammatory response including cellular infiltration, swelling and production of proinflammatory cytokines.

The skin microbiome is an important component of dermal immunoregulation [9]. Commensal microbes are known to protect from pathogens, and suggested to inhibit allergic sensitisation and inflammatory responses [10, 11], whereas unfavourable shifts in the microbiome are associated with certain skin diseases [9, 12]. In previous studies with skin models using OXA, most studies describe the OXA-induced changes in gut microbiome [13–15], and only one study describes the skin microbiome [16]. Therefore, not much is known about the effect of contact dermatitis on the skin microbiome, or the effect of OXA on microbiome in general.

In this study we characterised bacterial communities in murine OXA-induced CHS model to provide novel information of the skin microbiota during CHS inflammation. We evaluated the possible use of CHS model in questions related to inflammation-microbiome associations and study set-ups. We also carefully considered high contamination risk of CHS microbiota as well as other confounding factors, using appropriate bioinformatics tools to ensure reliability of our study.

## Materials and methods

### Mice

Female C57BL/6J mice (n = 72) were obtained from Envigo (Netherlands) and quarantined for one week. The mice were used in experiments at age of 6–7 weeks and were housed in transparent IVC plastic cages in groups of four, with each cage containing aspen chip bedding (Tapvei, Estonia). The mice were provided with standard mouse chow diet and tap water *ad libitum*. The laboratory animal facility had a 12 h dark-light cycle, a temperature of 20–21°C and relative humidity of 40–45%. The experiments were conducted in agreement with the European Convention for the Protection of Vertebrate Animals Used for Experimental and Other Scientific Purposes (Strasbourg March 18, 1986, adopted in Finland May 31, 1990). All experiments were approved by the State Provincial Office of Southern Finland (ESAVI/518/04.10.07/2017).

### Animal treatment protocol

A protocol for oxazolone-induced contact inflammation was used [17]. The mice were assigned into three groups (8 per group of which 4 per cage) and four timepoints. Oxazolone (OXA) was dissolved in a 4:1 (v/v) vehicle mixture of acetone and olive oil. During the sensitisation phase, the negative and positive mouse groups were administered with 50 μl of OXA (10 mg/ml) to the shaved and gently tape-stripped back skin under anaesthesia seven days before exposure (day -7). On the exposure day (day 0), the dorsal side of both mouse ears were challenged with 25 μl of OXA (3 mg/ml, positive group) or 25 μl of vehicle (acetone and olive oil only, negative group) solution. The mouse group which was both sensitised and challenged with OXA is called CHS positive group, and the mouse group which was only sensitised to OXA but challenged with a vehicle solution is called negative group (S1 Fig). The mice in the naïve group were not treated at all. After 24 hours, 7 days, 14 days or 28 days the mice were killed with isoflurane overdose. Biopsies were collected and stored at −80 °C for DNA extraction and microbiome analysis. For unknown reasons, one mouse died before the experiment.

### Histology

For the histological analysis, a piece of the ear biopsy was fixed in 10% buffered formalin and embedded in paraffin. 4 μm skin sections were cut and stained with haematoxylin and eosin (H&E) to measure epidermal and dermal thickness at 100 X magnification. The inflammatory cells (lymphocytes, neutrophils, and eosinophils) were counted in the skin viable layer, the dermis, from 4 μm HE stained skin sections with a light microscope under 1000 X magnification. Based on our previous publication [18], 15 high-power fields were selected for each mouse and the averages of the cell numbers from these fields were obtained to show the final number of each cell type per mouse. These inflammatory cells were recognised and counted based on their distinct differences in basic histology (e.g. structure, morphology, and colour). In brief, lymphocytes are mostly small in size and have a compact spherical nucleus with little visible cytoplasm; they are basophilic (pale blue/purple staining). Neutrophils have a multilobed nucleus (between 2 and 5 lobes) and stain a neutral pink. Eosinophils, looking larger than neutrophils, also have multiple lobes to their nucleus. In addition, they have large acidophilic specific granules in their cytoplasm that stain bright red or reddish-purple.

### RNA extraction and RT-PCR

Ear samples were homogenized in TRIsure reagent (Bioline Reagents Ltd., London, UK) using an Ultra-Turrax homogenizer, according to the manufacturer’s instructions. The concentration and integrity of extracted RNA was measured by NanoDrop spectrophotometer (ND-1000, Thermo Fisher Scientific Inc., Wilmington, NC, USA) and Bioanalyzer 2100 (Agilent, Santa Clara, United States), respectively. Complementary DNA (cDNA) was synthesised using Multi-Scribe Reverse Transcriptase and random primers (The High-Capacity cDNA Archive Kit, Applied Biosystems, Foster City, CA, USA) as described in our previous publication [19]. Primers and probes (18S ribosomal RNA, CXCL9, IFN-γ, IL-4 and IL-1β) for PCR analysis were ordered from Applied Biosystems. The PCR assays were performed in 96-well optical reaction plates with Relative Quantification 7500 Fast System (7500 Fast Real-Time PCR system, Applied Biosystems), following the manufacturer’s protocol.

### DNA extraction and sequencing

DNA extraction was adapted from [20, 21] using MasterPure Yeast DNA Purification Kit (Epicentre) and PureLink Genomic DNA Mini Kit (Invitrogen). Ear biopsy samples were incubated with 300 rpm shaking in 37 °C for 1 h in yeast cell lysis solution and 10,000 units of ReadyLyse Lysozyme solution. Samples were bead beaten with 0.5 mm zirconium beads (Sigma-Aldrich) in 6.5 m/s speed for 60 s and then incubated in 65 °C for 30 min with 1000 rpm shaking. Protein precipitation reagent was added, and the samples were centrifuged in 14,000 x g for 10 min. The supernatant was removed, mixed with isopropanol and transferred to PureLink Genomic DNA Mini Kit column, after which the kit’s instructions were followed. The DNA was eluted in 100 μl of the kit’s elution buffer. The samples were run in parallel to mitigate extraction batch bias.

The DNA was used as a template to amplify the V1-V3 regions of 16S rRNA genes by polymerase chain reaction (PCR) using pA (AGAGTTTGATCMTGGCTCAG) and pD’ (GTATTACCGCGGCTGCTG) primers with adapter sequences [22–24]. DNA input volume for thermal cycles was 1 μl, and the cycles started with 98 °C 30 s, then followed by 30 cycles of denaturation of 10 s in 98 °C, annealing of 30 s in 65 °C and extension of 15 s in 72 °C, followed by final extension of 5 min in 72 °C. Sequencing was done at the Institute of Molecular Medicine Finland (FIMM, University of Helsinki) with paired-ends of 2 x 300 bp with Illumina MiSeq. Control samples followed the same PCR protocols and included negative PCR controls and DNA extraction controls, the latter consisting of blank extractions with beads and extraction reagents only.

### Pre-processing of raw 16S rRNA gene sequences

PCR primer sequences were removed from the 16S rRNA gene sequences with Cutadapt (v. 2.7, [25]), followed by an initial quality analysis with MultiQC (v. 1.8, [26]). Further steps were performed in the R environment (v. 3.6.3). Due to the poor quality of reverse reads, only the 270 bp forward reads were used. With DADA2 package [27], the reads were filtered and trimmed with default parameters, sequence errors were removed with pseudo-pooling, and chimeras were removed from the generated sequence table with default parameters. The obtained amplicon sequence variants (ASVs) of 16S rRNA gene sequences were assigned taxa and species by comparing them to SILVA 132 databases [28]. Non-bacterial ASVs and ASVs which were unidentified on the kingdom level were removed. Contaminant ASVs were identified and removed using the “prevalence method” with threshold of 0.3 in the decontam R package [29].

One mouse sample was removed from the ASV data as the Shannon-index was extremely high compared to the number of sequences produced from these samples (i.e., library size) than what was observed in other samples. Cumulative sum scaling [30] and count normalisation methods [31] were tested for the sequences, however, library size variation was similar when the counts were not normalised. Since variation in library sizes across the remaining samples was consistent enough (min. 1336, max. 73345, mean 34764, median 35914), only the relative abundance transformation was used to equalise library sizes across samples.

### Statistical analyses

#### Evaluating cage effect

Cage effect describes a situation where certain microbes are strongly associated to individual cages, resulting in high similarity between corresponding samples, which could be confused to the effect being due to e.g., group or timepoint. Prior to statistics, cage effect was first explored by performing distance-based redundancy analysis (dbRDA), with which very high similarity of samples within a single cage was determined. To identify the ASVs of mice which were associated to certain cages, a generalised linear mixed model analysis was performed with negative binomial distribution with R package lme4 [32]. Each ASV was treated as the response variable, the interaction of group and timepoint as explanatory variables, while cage and mice were included as random effects, and natural logarithm transformed library sizes were added as an offset. The ASVs that were most associated with cage random effect (random effect variance > = 1) were removed from the dataset.

#### Phylogenetic tree

The most abundant ASVs (n = 100) were taken to construct a phylogenetic tree using FastTree (v. 2.1.11, [33]) using default parameters and aligned with SILVA 132 database.

#### Distance-based redundancy analysis (dbRDA)

Distance-based redundancy analysis (dbRDA) was performed using the R package vegan [34]. Relative abundances of each ASVs were used to calculate Bray-Curtis dissimilarity indices between samples as an input to a principal coordinates analysis (PCoA), and then running a redundancy analysis (RDA). Group and cage were used as explanatory variables, and DNA extraction batches as well as library sizes were treated as confounders and partialled out as conditioning variables. P values were calculated with ANOVA like permutation test for dbRDA (permutations = 9999) pairwise comparisons using biodiversityR package [35].

#### Hierarchical modelling of species communities

The most abundant ASVs (n = 100) were chosen for hierarchical modelling of species communities (HMSC) analysis, which is a class of joint-species distribution modelling [36, 37]. Using the R package HMSC [38], ASVs were included as response variables, and the interaction of group and timepoint as an explanatory variable, while mice and cages were included as random effects. Bayesian inference was used to fit the sequence count data to a lognormal Poisson model. The naïve group and timepoint were treated as baselines. The model was thinned to every 100th iteration and run for 107,500 Markov Chain Monte Carlo iterations, out of which 7500 were discarded as transient, i.e., not considered for subsequent analyses. Variance partitions were created to identify for which ASVs the interaction of group and timepoint accounted for most variation.

#### Correlation analysis

Spearman Rho correlations tests [39] with FDR corrections for statistical significance were calculated between epidermis and dermis or immune cell counts and ASV abundance and visualised using R package ggplot2 [40] and GraphPad Prism (v. 9.3.1, GraphPad Software, San Diego, California USA, www.graphpad.com).

#### Other statistical tests

Difference in epidermis and dermis thickness between groups were calculated with Brown-Forsythe and Welch ANOVA and Dunnett’s T3 multiple comparison tests and difference of immune cell counts between groups cells were calculated with Kruskal-Wallis and Dunn’s multiple comparison tests in GraphPad Prism.

Differences between 15 most abundant bacterial families were calculated using the R package DESeq2 [31] differential abundance analysis that applies a generalised linear model with negative binomial distribution and FDR correction for multiple comparisons.

Post analysis statistical tests of HMSC were done using a generalised linear model with negative binomial and zero-inflated log-normal distributions, with R packages DESeq2 and MetagenomeSeq, respectively [30, 31]. FDR correction for multiple comparison was included. The purpose of these tests was to compare results of HMSC using Bayesian inference to the classical frequentist inference that are familiar with most readers.

## Results

### Oxazolone induced inflammation appeared strongest in skin a day after the exposure

**Fig 1:**
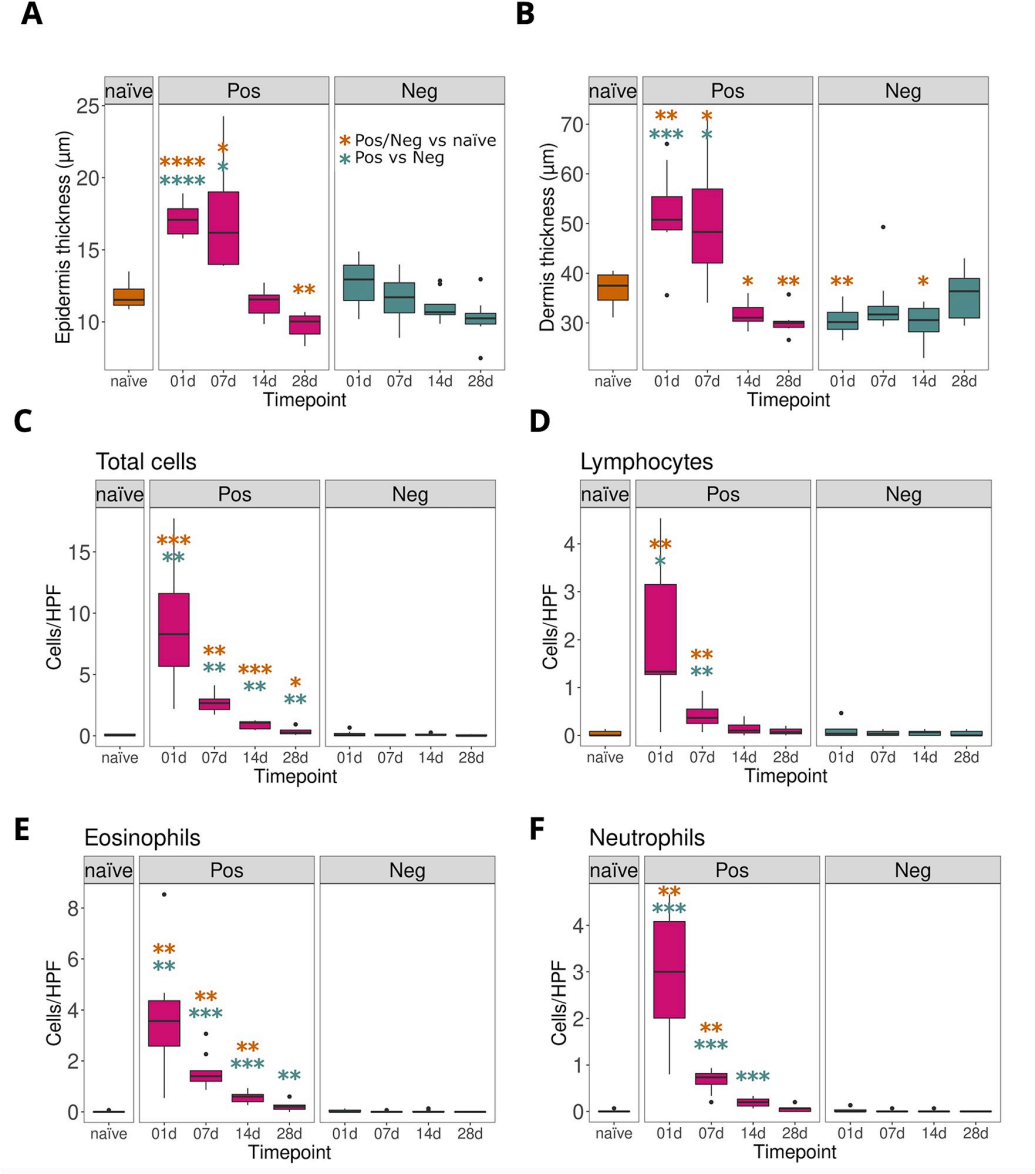
Repeated exposure to oxazolone induces skin thickening and recruitment of immune cells. OXA-induced inflammation was studied by measuring thickness of epidermis (A) and dermis (B) in H&E-stained skin sections. The number of total cells (C), lymphocytes (D), eosinophils (E) and neutrophils (F) were counted from H&E-stained skin tissue under a light microscope at 1000x magnification. P values for epidermis and dermis were calculated with Brown-Forsythe and Welch ANOVA and Dunnett’s T3 multiple comparison tests and P values for immune cells were calculated with Kruskal-Wallis and Dunn’s multiple comparison tests. Yellow significance stars indicate the test between naïve and positive or naïve and negative, and blue significance stars between negative and positive group. P values: * < 0.05. ** < 0.01, *** < 0.001, **** < 0.0001.

To observe markers for inflammation, skin thickness and immune cell counts were determined. In the positive group, the second OXA exposure resulted in a statistically significant thickened layer of epidermis and dermis after 1 and 7 days of exposure (Fig 1A and 1B, S2A and S2B Fig). On day 14, skin thickening of the positive group diminished. The skin thickness of mice in negative and naïve groups remained consistent and no thickening was observed during the follow-up of 28 days. Immune cells were counted from the haematoxylin and eosin (H&E) -stained skin sections to further measure inflammation, namely the number of total cells, lymphocytes, eosinophils, and neutrophils (Fig 1C–1F). All cell counts were statistically elevated in positive group on day 1 and were still elevated on day 7 when compared to naïve or negative group. Counts were low in both negative and naïve group during the follow-up. The gene expression of IFN-γ, IL-4, IL-1β and CXCL9 were also highly enhanced in the positive group at day 1 when compared to negative or naïve mouse groups (S3 Fig).

### Skin microbiota of laboratory mouse included bacterial taxa typically occurring in skin and gut

16S rRNA genes were sequenced from mouse ear biopsies to determine the skin microbiome. After sequence pre-processing, a total of 1650 ASVs remained for subsequent steps. To show the common bacterial taxa and their strain-level variability, a phylogenetic tree was constructed for 16S rRNA gene sequences. One major branch in the phylogenetic tree showing the most abundant ASVs constituted of the *Muribaculaceae* family (S4 Fig). The family had the most variation in bacterial strains. Other branches included taxa typically occurring in the skin such as *Streptococcaceae* and *Staphylococcaceae* and gut associated bacteria like *Erysipelotrichaceae* and *Lachnospiraceae*.

**Fig 2:**
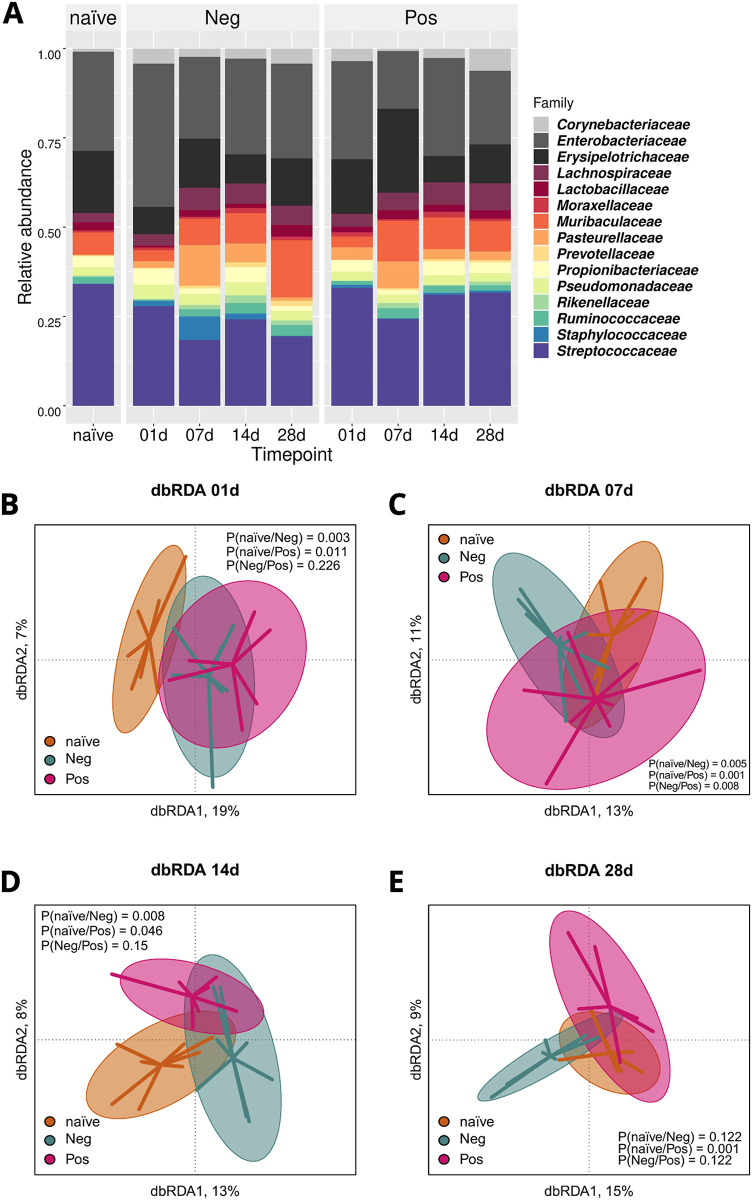
Changes in mouse skin microbiota were induced by acetone and olive oil with and without oxazolone. The most abundant bacterial families (n = 15) were chosen for visualisation using their relative abundances in the communities (A). Distance-based redundancy analysis (dbRDA) was performed for each timepoint to observe microbial community differences between the groups over time (B-E). Relative abundance data was used to calculate Bray-Curtis dissimilarity indices between samples with group and cage as categorical explanatory variables, while the ASVs were the response variables. In addition, DNA extraction batches and library sizes were included as conditioning variables and partialled out. Ellipses on the top of symbols mark the 85% confidence interval and the P values of ANOVA like permutation test for dbRDA (permutations = 9999) pairwise comparisons are included in each box.

Visualisation of microbial relative abundances showed families of *Enterobacteriaceae*, *Erysipelotrichaceae*, *Muribaculaceae*, and *Streptococcaceae* to be abundant in all groups and timepoints (Fig 2A). Other bacterial families which were present in all groups in varying degrees of abundance included families such as *Corynebacteriaceae*, *Lachnospiracecae*, *Pasteruellaceae*, *Prevotellaceae* and *Propionibacteriaceae*. *Enterobacteriaceae* were most abundant in the negative group on day 1. On day 7, *Erysipelotrichaceae* and *Pasteurellaceae* were most abundant in the positive and negative group, respectively, and the negative mice had less *Muribaculaceae* compared to other timepoints. *Streptococcaceae* were relatively abundant in the naïve group, while *Pasteurellaceae* were not. *Pasteurellaceae* and *Erysipelotrichaceae* had statistically significant differences mostly between positive and naïve groups (S1 Table), even though individual mouse variation could be observed (S5 Fig). The differences of abundances on days 1 and 7 indicated differences between groups and timepoints and were further shown with successive statistical tests.

### Oxazolone induced change in the microbial community was observed seven days after exposure

Before studying the effect of OXA on skin microbiome, a linear model was used to identify if the mouse cages had a skewing effect on the microbiome. Three ASVs were identified to be significantly associated with distinct cages, thus not representing the effect of OXA, and removed from the dataset (S6 Fig). The difference in microbiotas between positive and negative group, induced by OXA, was statistically significant on day 7 (P = 0.008), specified by distance-based redundancy analysis (dbRDA, Fig 2). Subsequently, the microbial community composition of both positive and negative groups significantly differed from the microbiota of naïve group on day 1 (Fig 2B). The difference, which was induced by vehicle exposure of acetone and olive oil, remained significant up until day 14 (Fig 2C and 2D). On day 28, only the positive group differed from the naïve group (Fig 2E). The timepoints showed statistically significant differences within the positive group, but not within the negative group (S7 Fig).

### Gut-associated taxa were observed in the skin of mice after one and seven days of oxazolone exposure

**Fig 3:**
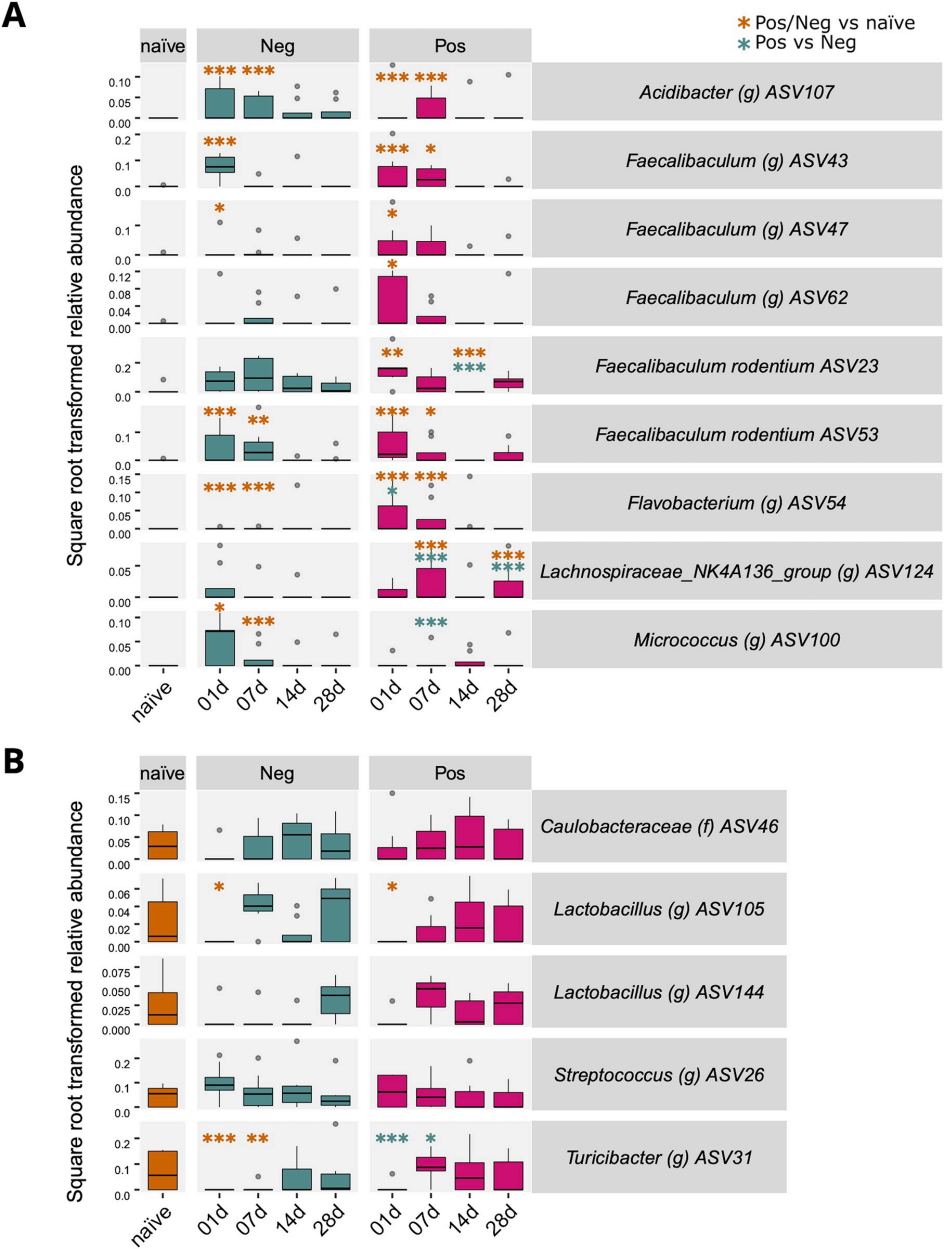
Microbial abundance in different groups and timepoints based on variance partition analysis. Hierarchical Modelling of Species Communities (HMSC) model was utilised for the most abundant ASVs (n = 100) to examine changes of ASV abundance between groups and timepoints. ASV abundances were included as a response variable, while the interaction of group and timepoint in addition to library size were included as explanatory variables to model the communities. Each mouse and cage were included as random effects. The ASVs with the most variance originating from the interaction of positive group and timepoint (A) and of negative group and timepoint (B) are shown. The P values were calculated using differential abundance analysis using generalised linear models with zero-inflated log-normal and negative binomial distribution, including FDR correction. Yellow colour indicates the test between naïve and positive or naïve and negative, and blue between negative and positive group: * < 0.1, ** < 0.01, *** < 0.001.

A Bayesian model, Hierarchical Modelling of Species Communities (HMSC), and a classical generalized linear model, producing P-values, were used to study the differences in abundances of ASVs between the groups and timepoints (Fig 3). The ASVs that varied the most in the positive group between each timepoint, such as ASV43 representing the genus *Faecalibaculum*, were nearly absent in the naïve group (Fig 3A). Most of these ASVs were less abundant in the negative group than in the positive group, especially on days 1 and 7. The difference between negative and positive groups highlighted the effect of OXA. These abundant ASVs of the positive group mostly belonged to the genus *Faecalibaculum*, which can typically be found in the gut. On the contrary to the ASVs varying in the positive group (Fig 3A), ASVs varying most in the negative group were present in the naïve group (Fig 3B). These ASVs were nearly absent in the negative and positive groups on day 1, except for ASV46 *Caulobacteraceae* and ASV26 *Streptococcus*, suggesting the effect of vehicle exposure. The difference of ASV abundances were statistically significant especially when comparing either positive or negative group with the naïve group on days 1 and 7. In addition, differences between timepoints either within the positive or negative group were statistically significant on many occasions (Table 1).

**Table 1:** Pairwise statistical test results of microbial abundance of within group timepoints based on HMSC. Each ASV, its lowest rank, statistical test, comparison, and the result are given in each column. GLMM = generalised linear mixed model.

ASV	Lowest rank	Test	Variable	Comparison	P value
ASV107	*Acidibacter*	NB	Pos	01d vs 14d	***
		NB	Pos	07d vs 14d	***
ASV43	*Faecalibaculum*	NB	Pos	01d vs 14d	***
		NB	Pos	07d vs 14d	***
		NB	Pos	14d vs 28d	***
ASV62	*Faecalibaculum*	NB	Pos	01d vs 14d	***
		NB	Pos	07d vs 14d	***
		NB	Pos	14d vs 28d	***
ASV23	*Faecalibaculum rodentium*	ZI	Pos	01d vs 14d	***
		NB	Pos	01d vs 14d	***
		NB	Pos	07d vs 14d	***
		NB	Pos	14d vs 28d	***
ASV53	*Faecalibaculum rodentium*	ZI	Pos	01d vs 14d	*
		NB	Pos	01d vs 14d	***
		NB	Pos	07d vs 14d	***
		NB	Pos	14d vs 28d	***
ASV54	*Flavobacterium*	NB	Pos	01d vs 28d	***
		NB	Pos	14d vs 28d	***
ASV105	*Lactobacillus*	ZI	Neg	01d vs 07d	***
		NB	Neg	01d vs 07d	**
		NB	Neg	01d vs 28d	**
ASV144	*Lactobacillus*	ZI	Neg	01d vs 28d	*
ASV26	*Streptococcus*	NB	Neg	01d vs 28d	**
ASV31	*Turicibacter*	NB	Neg	01d vs 07d	***
		NB	Neg	01d vs 14d	***
		NB	Neg	01d vs 28d	***

NB, negative binomial generalized linear model

ZI, zero-inflated log-normal mixture model

### Gut and oral -associated bacteria correlate with inflammation markers

**Fig 4:**
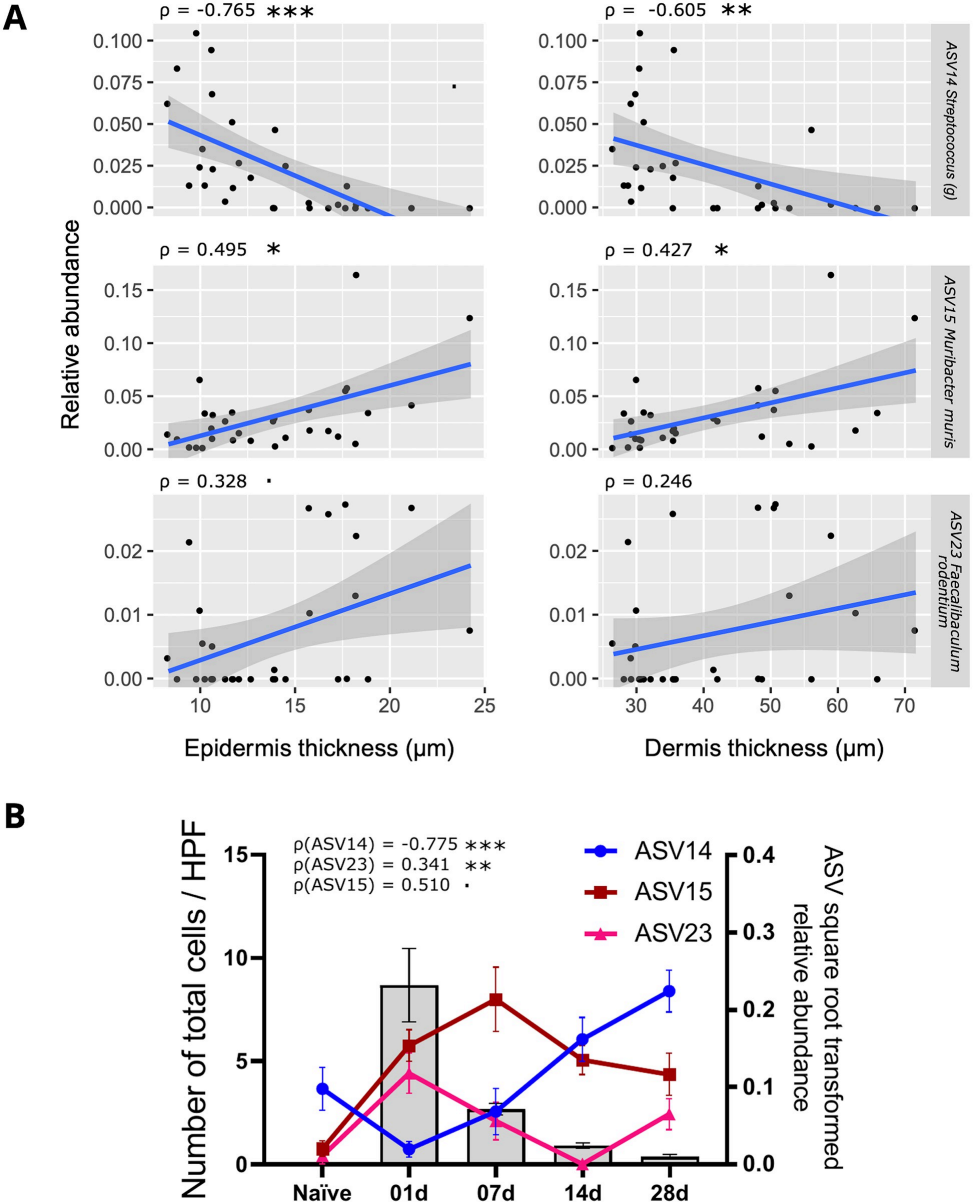
Associations between skin thickness or immune cell recruitment and ASVs. Positive group Spearman correlations were calculated between ASV abundance and epidermis/dermis thickness (A) as well as total immune cell counts (B). Cells were counted in 15 high-power fields at 1000 X magnification. Spearman Rho rank correlation test with FDR correction was used to calculate P values: < 0.1, * < 0.05, ** < 0.01, *** < 0.001.

Pairwise Spearman correlations were calculated between ASVs and skin thickness or immune cell counts (Fig 4). Within positive group, ASV14 representing *Streptococcus*, typical in skin, correlated negatively with skin thickness and immune cells (P < 0.01 for epidermis and dermis, P < 0.001 for total cell count). Positive correlations between ASVs and immune cells or skin thickness were observed for ASV15 *Muribacter muris* (P < 0.05 for epidermis and dermis, P < 0.01 for total cells) and for ASV23 *Faecalibaculum rodentium* (P < 0.1 for total cells), often found in oral and gut microbiome, respectively. When considering naïve group as a starting point, ASV14 *Streptococcus* relative abundance declined on day 1 and slowly increased until day 28. On the contrary, the relative abundance of ASV15 *Muribacter muris* and ASV23 *Faecalibaculum rodentium* increased on day 1, with ASV15 *Muribacter muris* reaching its peak on day 7 and then decreasing. Similar patterns were observed with lymphocytes, eosinophils and neutrophils (S8 Fig)

## Discussion

We characterised the skin microbiota in the mouse model of contact dermatitis and evaluated its possible use for skin host-microbe interaction studies. In our oxazolone-induced CHS model, the inflammatory reaction in the positive CHS group peaked at 24 h and remained high until day 7 after hapten re-exposure, resulting in skin thickening observed both in epidermis and dermis, as well as an influx of immune cells. Also, the gene expression of Th1-prone IFN-γ, Th2-cytokine IL-4 as well as pro-inflammatory IL-1β and chemokine CXCL9, which attracts neutrophils, were significantly increased, especially at day 1. At the same time no consistent responses were observed in control groups. Other studies have also shown that oxazolone does not induce pure Th1-response but instead mixed immune response involving both T1 and T2-branches of the adaptive immunity as well as proinflammatory innate responses [41, 42]. Therefore, our model worked as expected and is in line with the previous studies [3, 43].

Characterising the skin microbiota of laboratory mice helps interpret inflammation-microbiota associations. By exploring the taxa from the naïve group, inflammation induced changes in the microbiota can be described, and confounding effects such as changes related to the cage environment itself can be identified. In addition to skin-associated taxa like *Streptococcus*, *Staphylococcus* and *Corynebacterium* [9], we observed several gut-associated taxa such as *Faecalibaculum* and *Muribaculaceae* [44, 45] on the mouse skin. Many gut-associated families such as *Erysipelotrichaceae*, *Enterobacteriaceae* and *Muribaculaceae* were also observed in the untreated naïve group, suggesting that gut-associated microbes are a normal part of laboratory mouse skin microbiome. This is likely due to the mice living in a confined space, where they are constantly exposed to faecal matter of their own and their fellow mice in the same cage. Additionally, mice are coprophagic and faecal matter can therefore be transferred on mouse skin through grooming. Similarly, recent studies have observed gut-associated taxa on laboratory mouse skin [46, 47]. In the same studies wild mice had less gut-associated taxa on their skin compared to laboratory mice. Based on these previous and current studies, animal facilities influence the skin microbiome.

Comparing microbial changes between the experimental groups can help determine which changes are induced by the modelled contact dermatitis or other treatments such as vehicle exposure. In addition, microbial differences caused by factors such as DNA extraction batch can be perceived to be the result of experimental procedures. We considered these confounders, including DNA extraction batch, cage effect and contamination, with appropriate sampling and extraction protocols, as well as with our analyses. Regardless of these considerations, significant between-group differences were harder to detect on family and ASV level, since abundances of bacteria varied notably between individuals. Additionally, the ASVs had low abundance, which suggests the changes aren’t limited to single ASVs, but rather to communities. When comparing changes on the microbial community level, the negative group differed from the naïve group already on day 1. Furthermore, on ASV level, the bacteria which were present in the naïve group but absent in the negative group were mostly absent on day 1 only. Vehicle exposure with acetone and olive oil could explain the difference in microbiota between the negative and naïve group, since the naïve group was not treated at all. Antimicrobial effect of both acetone and olive oil have been reported [48, 49], additionally the vehicle solution could promote some bacteria that effectively utilise fatty acids in the olive oil. Local changes in microbial community induced by vehicle solution have also been demonstrated with ethanol [16]. However, with our study setup we cannot fully exclude the effect of OXA given during sensitisation phase. It is well known that the hapten-driven immunological changes in the host are systemic, they can induce local inflammatory responses after the re-encounter of the hapten molecules also in locations other than the original skin sites via causing changes in skin pH, available nutrients, or salt concentration in addition to inducing swelling of outermost skin layers, enhanced circulation, and inflammatory cell recruitment. Similarly, to our knowledge systemic effects have not been reported on host skin after acetone, ethanol or olive oil exposure. Vehicle effect was already and mostly observed on day 1, which suggests the effect occurred in hours.

In our study, microbiota seemed to respond to OXA-induced inflammation more slowly than to the vehicle solution. Since both experimental groups were treated similarly with OXA during the sensitisation phase, it is probable that the seen differences between naïve and experimental groups are due to the vehicle given during the challenge in the negative group and due to the vehicle and hapten-induced inflammatory response in positive group. On microbial community level, the difference between positive CHS group and negative group was statistically significant on day 7, which demonstrated the effect of OXA on the microbiota. This is in line with the inflammatory reaction, which was significantly enhanced at days 1–7 after hapten re-exposure in the positive CHS group. In addition, microbial communities differed between timepoints in the positive group but not in the negative group, which further supported the effect of OXA. OXA effect on microbiomes has not been widely explored. Previous studies related to microbiomes with OXA exposure include OXA-induced atopic dermatitis and colitis. Studies on colitis models either describe the gut microbiome on a community level or not at all, although one study describes the microbiome on the family level [50–53]. With OXA-induced atopic dermatitis models, most studies explore the gut microbiome, and one study explores the skin microbiome [13–16]. In the study examining the skin microbiome of an OXA-induced atopic dermatitis model by [16], OXA treatment resulted in somewhat similar effects on the microbiota. In their NMDS ordination analysis, the control, vehicle and OXA treated groups were differently distributed compared to each other. However, the authors did not specify the significance of pairwise comparisons of these groups, therefore, direct comparison is with our study is difficult. In the same study, OXA treatment reduced bacterial richness compared to controls. We did not observe reduction in richness, which is possibly due to different mouse models and therefore differing OXA exposure protocols, with our study having only one OXA challenge compared to six in the study by [16]. Additionally, it is possible that microbiome changes in CHS do not contribute to the skin condition as much as with e.g., atopic dermatitis. Here, when considering shifts in microbiota between groups treated with or without OXA, it is evident that OXA and the resulting skin inflammation altered the microbial community. The effect was slower compared to vehicle effect and induced community changes that were not resolved in 28 days.

On ASV level, most of the bacteria that were present in the positive group but absent in the naïve or negative group belonged to the genus *Faecalibaculum*. At the time of writing, only one species of this genus has been recorded in the List of Prokaryotic names with Standing in Nomenclature (https://lpsn.dsmz.de/genus/faecalibaculum), which was *Faecalibaculum rodentium*. *F*. *rodentium* is an obligate anaerobe Gram-positive bacterium and was isolated from C57BL/6J mice faeces [44]. Similarly, many of the skin-associated taxa such as *Streptococcus* and *Staphylococcus* are Gram-positive. *Faecalibaculum* bacteria, however, cannot utilise oxygen, which could explain why we observed *Faecalibaculum* on the skin for a short period, mostly on days 1 and 7. As mouse faecal bacteria, *Faecalibaculum* bacteria were already present in the cage environment, and skin inflammation possibly created a more suitable environment, allowing *Faecalibaculum* bacteria to act as opportunists. Alternatively, since 16S rRNA gene sequencing cannot distinguish the physiological status of the microbes, *Faecalibaculum* might have accumulated on the skin as dead cells.

Identifying associations between skin inflammation and microbiota can help characterise the nature of host-microbiota interactions. We found ASVs representing *Streptococcus*, *Muribacter muris* and *Faecalibaculum rodentium*, and their abundance either increased or decreased along with immune cell counts and epidermis or dermis thickness. Both skin thickness and immune cell counts indicated skin inflammation, thus similar correlation profiles were consistent. Abundance of *Streptococcus*, which could be considered a native skin bacterium, correlated negatively with both immune cell counts and epidermis and dermis thickness. *Streptococcus* abundance lowered during inflammation peak on day 1, and slowly increased thereafter. In contrast, the abundances of *M*. *muris* and *F*. *rodentium* increased on day 1 and correlated positively with inflammation. Interestingly, *M*. *muris* is an oral bacterium first isolated from mice [54, 55] and its temporal abundance profile was almost opposite compared to *Streptococcus*. Dermal inflammation likely caused itch, leading to the mice grooming themselves more, which in turn could have transferred a higher abundance of *M*. *muris* on the skin at the expense of *Streptococcus*. Both our correlation analysis and HMSC analyses seemed to indicate that CHS inflammation created more convenient conditions for bacteria which are not typical on the skin, taking the space from native skin bacteria.

As a conclusion, CHS mouse model is reliable for immunological studies, and based on our results, it could be used to study changes induced by contact dermatitis in the skin microbiome, with considerations that should be addressed. In addition to the vehicle solution used to administrate the hapten, other confounding factors include cage effect and DNA extraction batches, which can be controlled by careful sampling of controls, decontamination methods as well as comprehensive statistical analyses. A major contributor to laboratory mouse skin microbiome seems to be the animal facility, and the used model itself. Despite the challenges, we have demonstrated that OXA-induced contact dermatitis does result in changes in the microbial community, which very probably cannot be explained by confounding effects, such as the vehicle effect. We found that skin inflammation created a favourable environment for certain bacteria to act as opportunists, in our case *Faecalibaculum* and *Muribacter muris*, which was disadvantageous for native skin bacteria such as *Streptococcus*. Therefore, CHS model could be used in studies exploring the ecological benefits of inflamed skin for different bacteria.

## Supporting information

S1 Fig Schematic of the experiment. (TIFF)

S2 Fig Histological ear tissue stainings. (TIFF)

S3 Fig Gene expression of selected cytokines and chemokines. (TIFF)

S4 Fig A phylogenetic tree of the microbiota reveals taxa commonly found in the skin and gut. (TIFF)

S5 Fig Individual variation of the microbiota observed on family level. (TIFF)

S6 Fig Demonstrating the cage effect. (TIFF)

S7 Fig Distance-based redundancy analysis of different timepoints within the groups. (TIFF)

S8 Fig Associations between lymphocytes, neutrophils and eosinophils and ASVs. (TIFF)

S1 Table Pairwise statistical tests of the 15 most abundant families. (TIFF)
