# Supplementary figures and images for: Skin microbiota of oxazolone-induced contact hypersensitivity mouse model
Source: PLoS One. 2022 Oct 20;17(10):e0276071. doi: 10.1371/journal.pone.0276071 (PMC9584374; doi:10.1371/journal.pone.0276071)

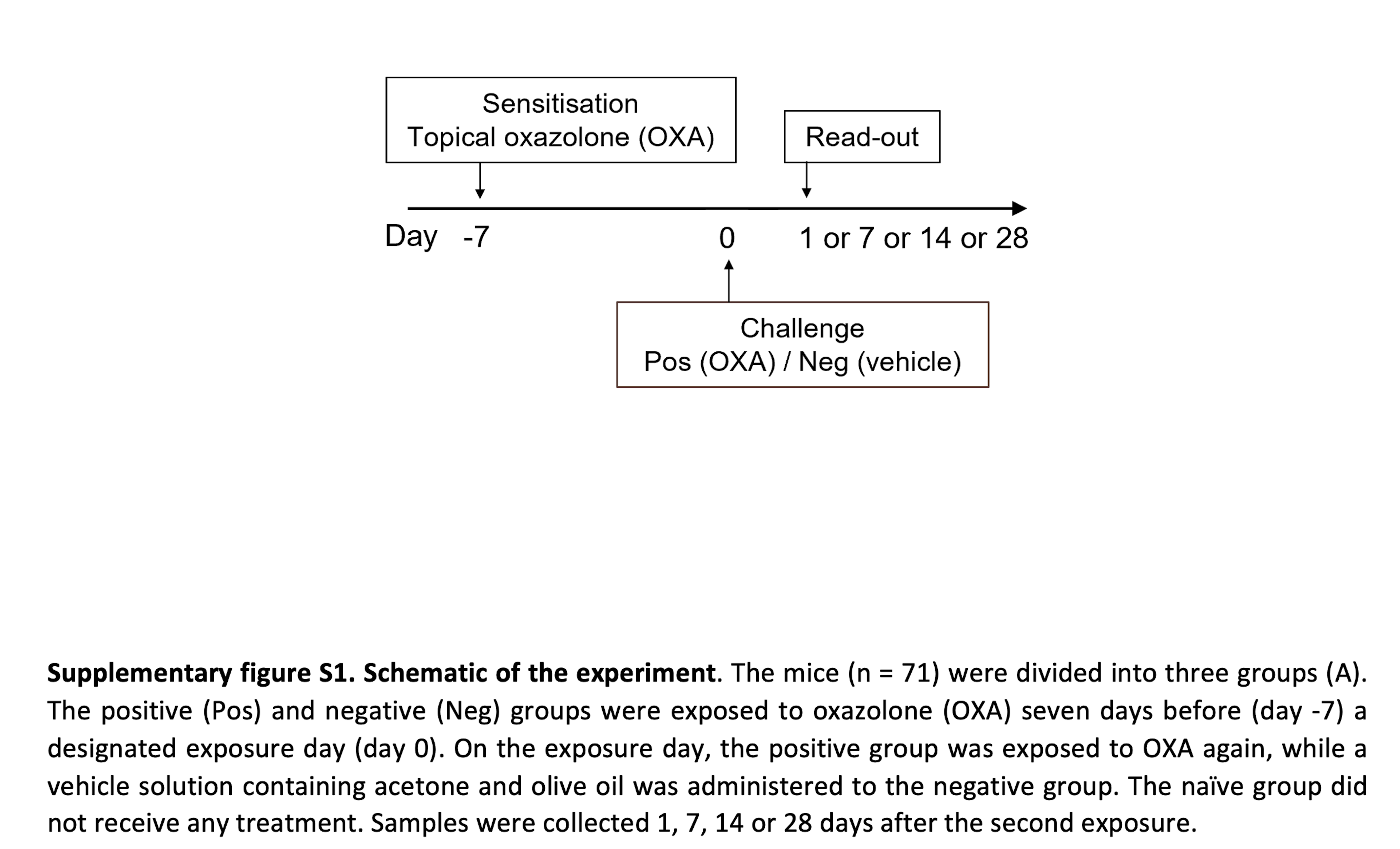

Supplement: S1 Fig — (TIFF) [file pone.0276071.s001.tiff]

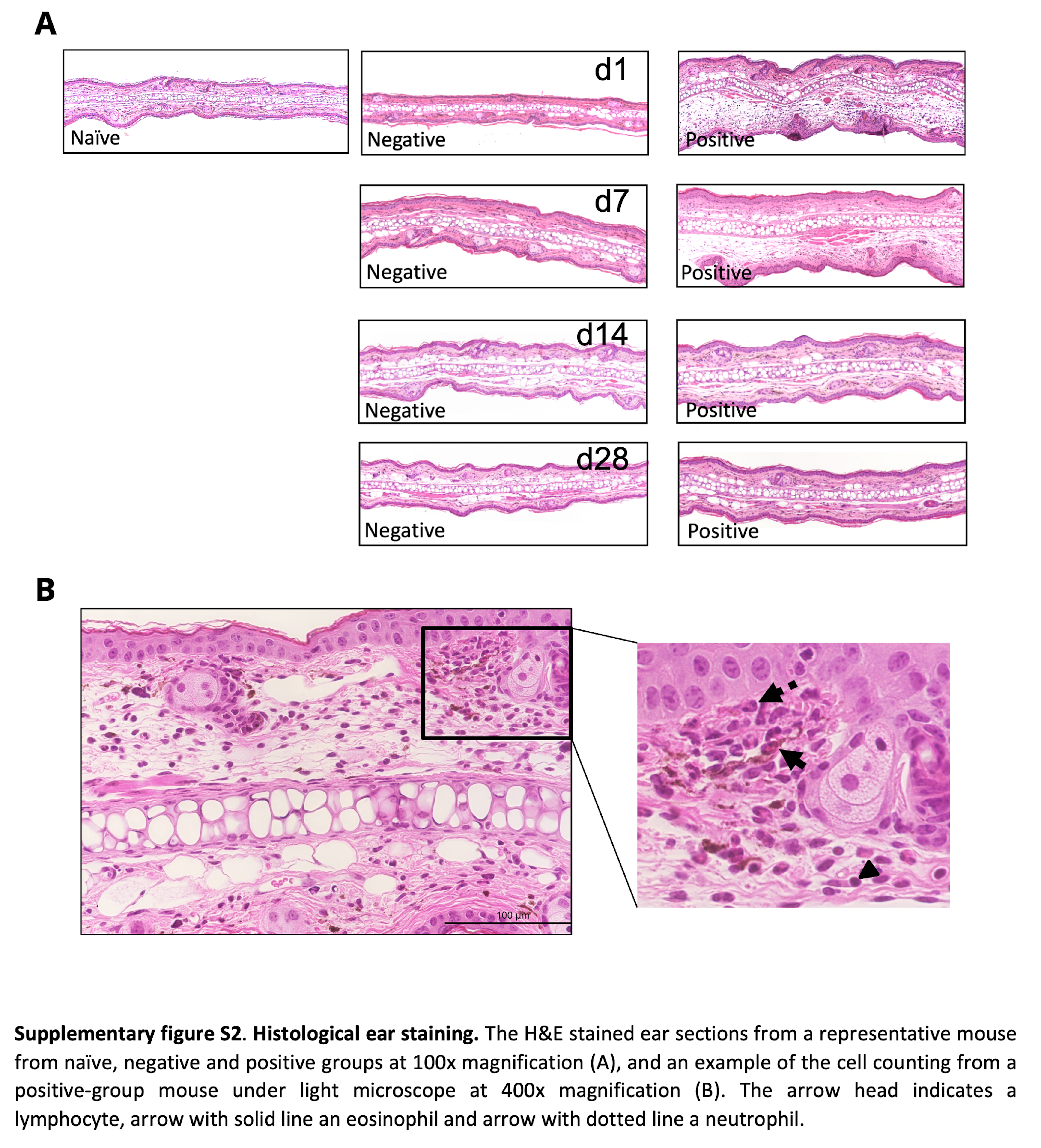

Supplement: S2 Fig — (TIFF) [file pone.0276071.s002.tiff]

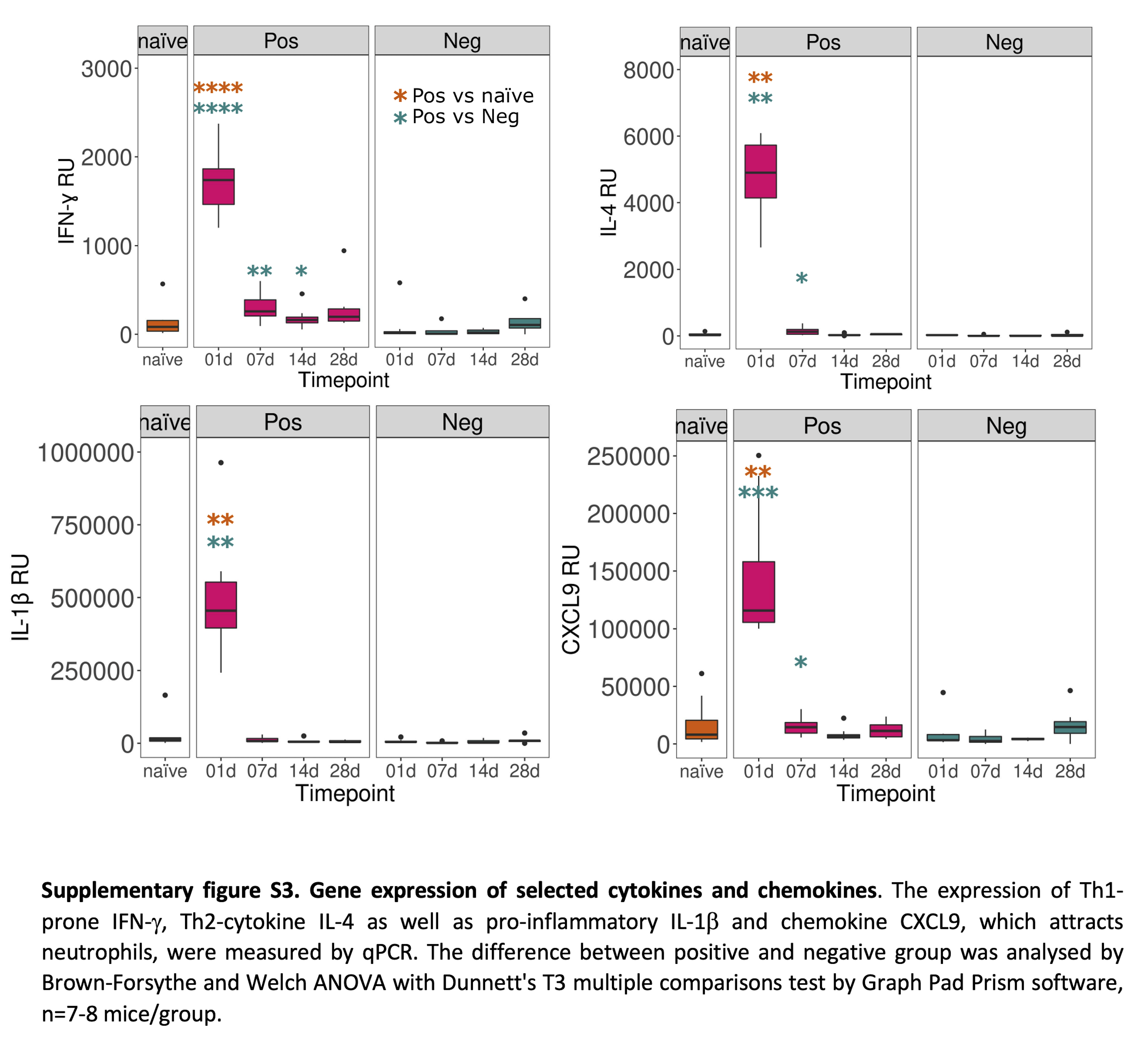

Supplement: S3 Fig — (TIFF) [file pone.0276071.s003.tiff]

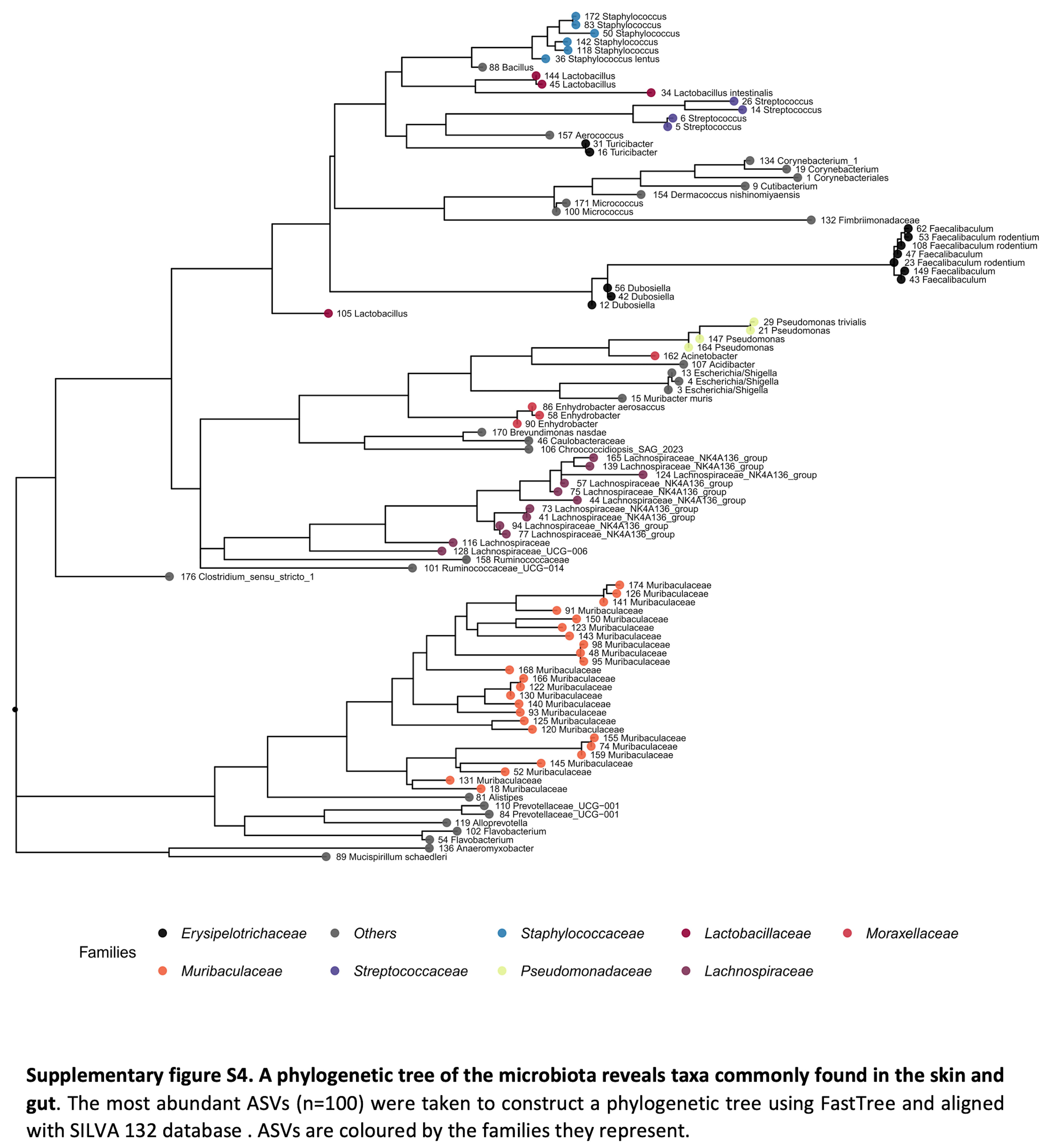

Supplement: S4 Fig — (TIFF) [file pone.0276071.s004.tiff]

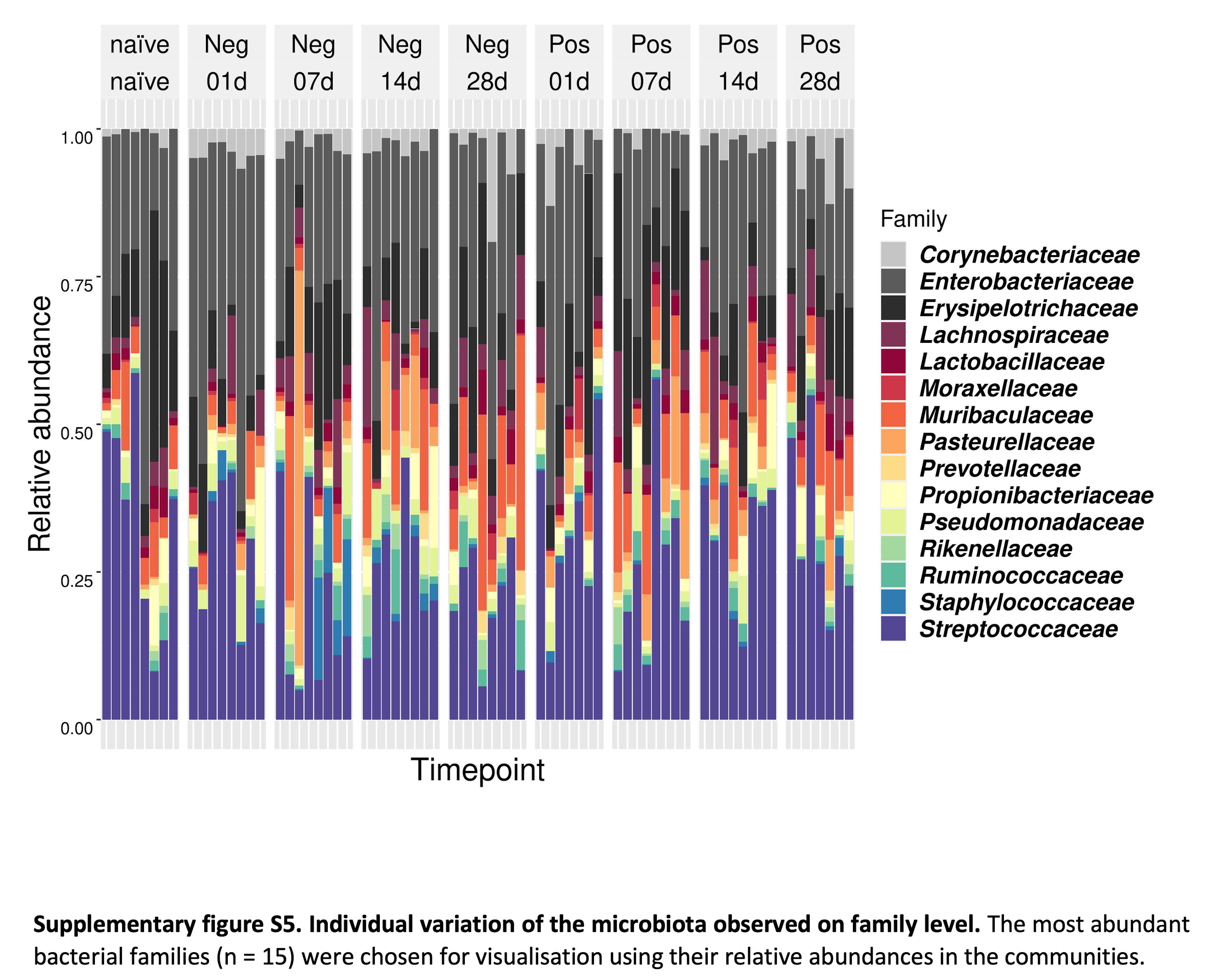

Supplement: S5 Fig — (TIFF) [file pone.0276071.s005.tiff]

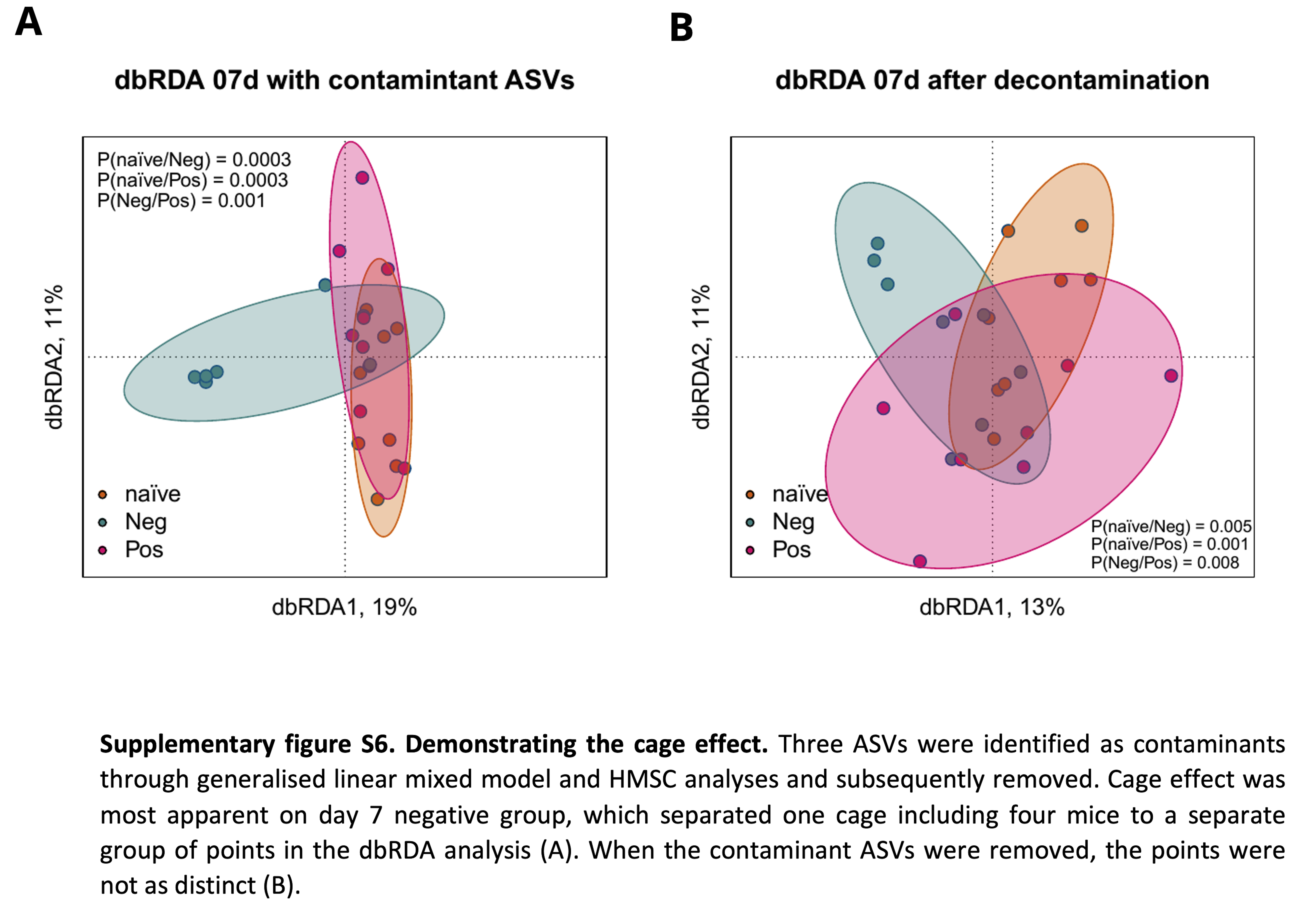

Supplement: S6 Fig — (TIFF) [file pone.0276071.s006.tiff]

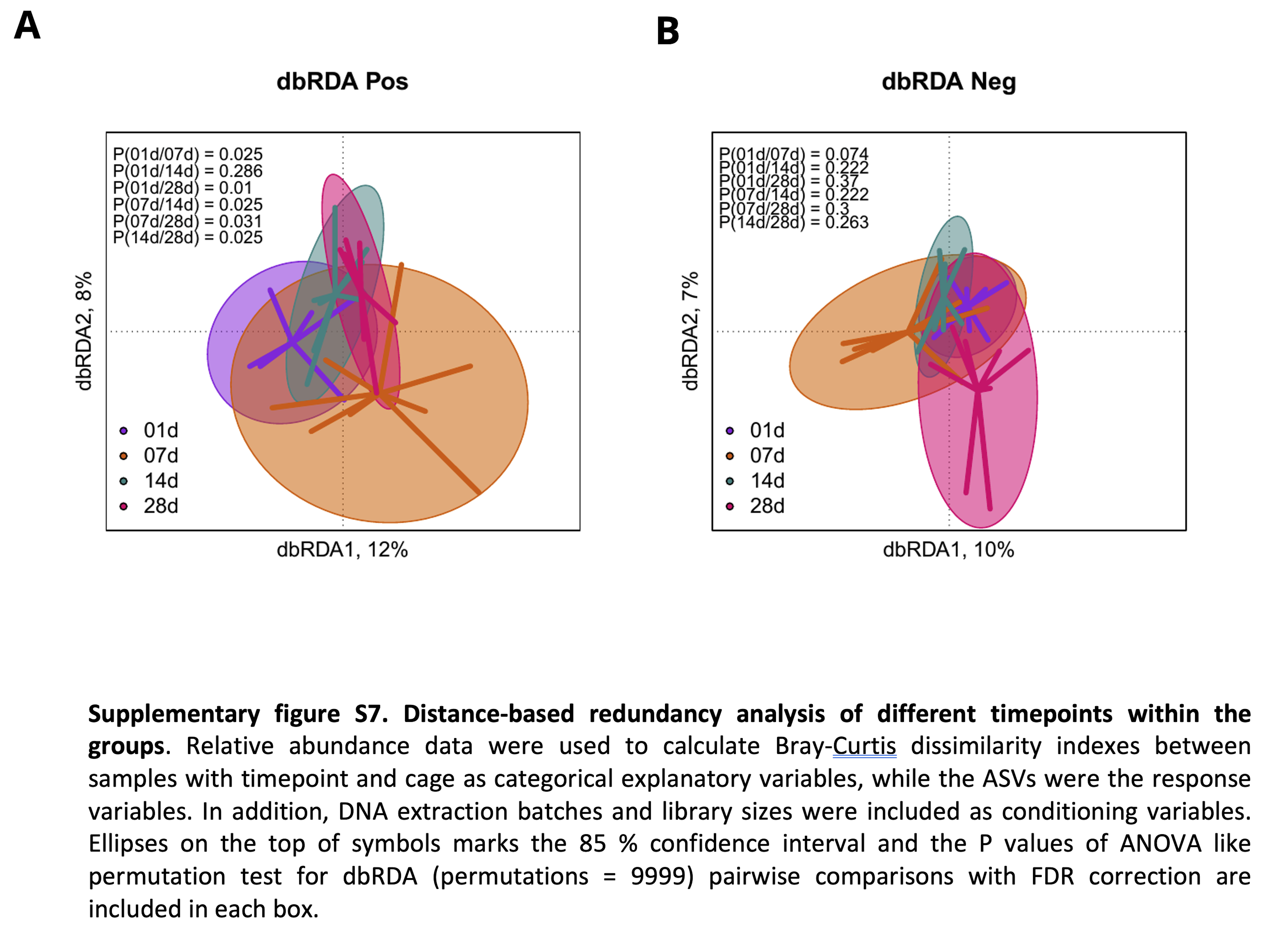

Supplement: S7 Fig — (TIFF) [file pone.0276071.s007.tiff]

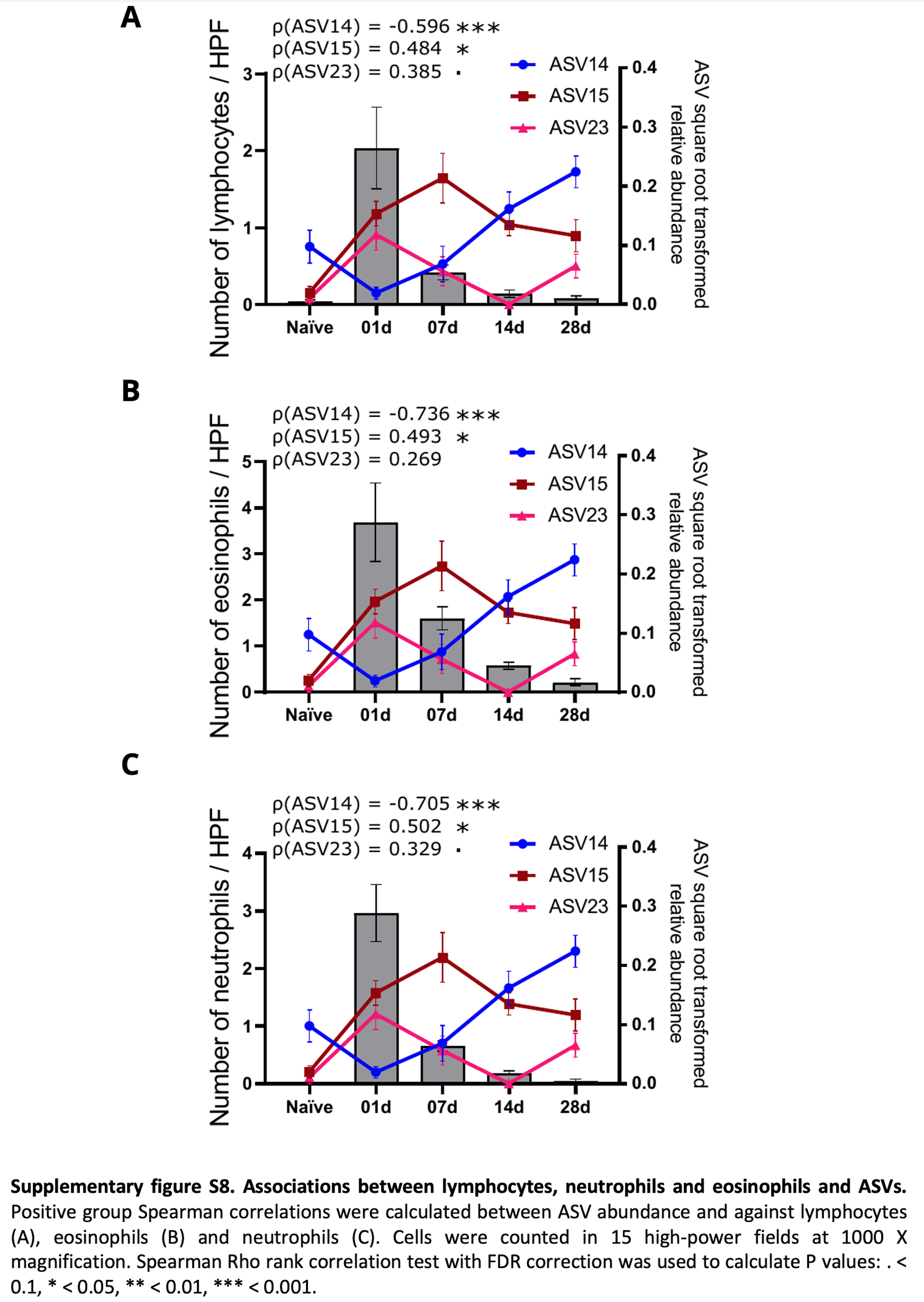

Supplement: S8 Fig — (TIFF) [file pone.0276071.s008.tiff]

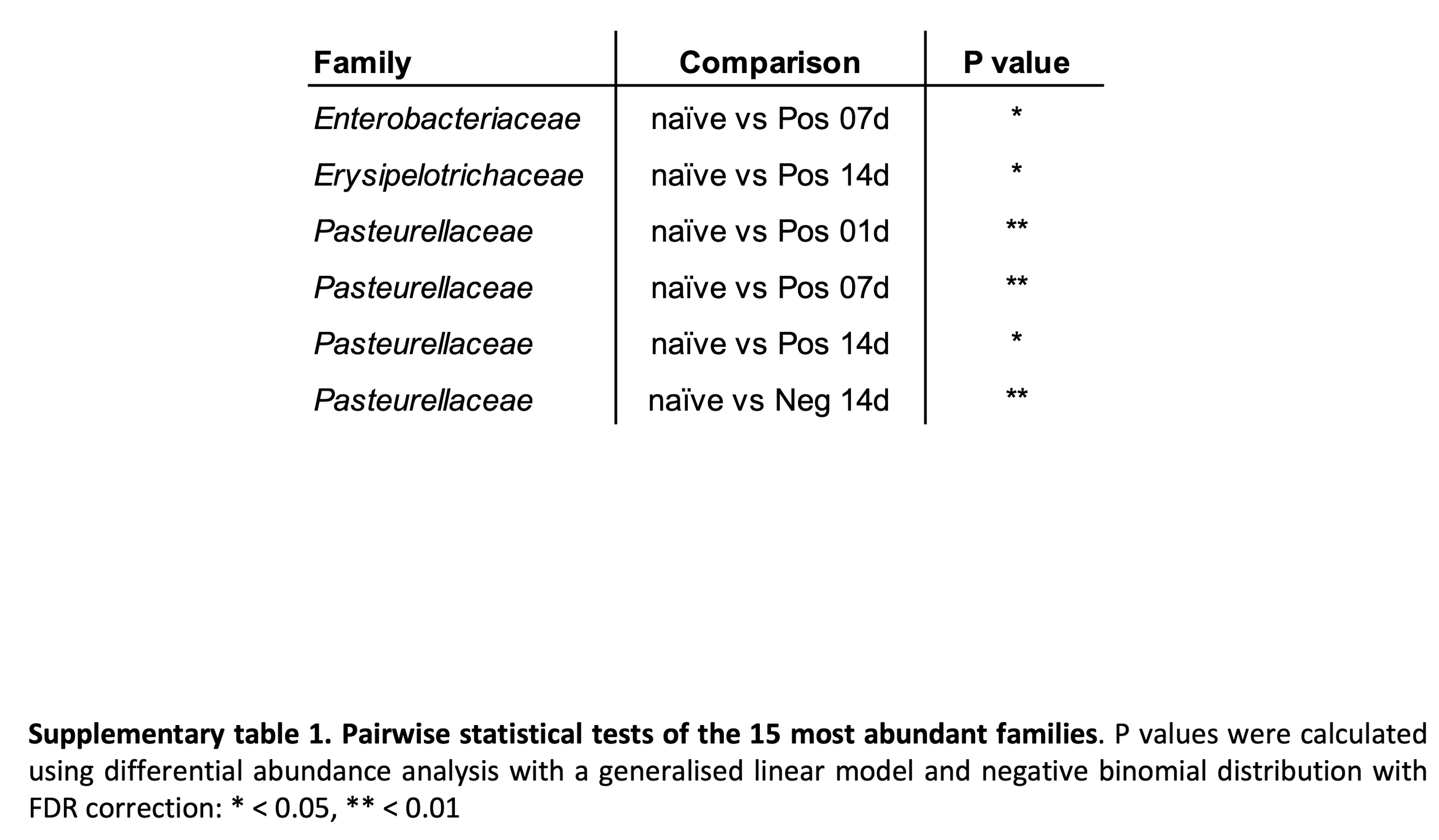

Supplement: S1 Table — (TIFF) [file pone.0276071.s009.tiff]
